# Critical Design and Operating Parameters of Active Waveguide Bragg Gratings for Laser Performance †

**DOI:** 10.3390/mi15121468

**Published:** 2024-11-30

**Authors:** Ángel Sanz-Felipe, Juan A. Vallés

**Affiliations:** 1 Applied Physics Department, Escuela Politécnica Superior, University of Zaragoza, Ctra. de Cuarte s/n, 22071 Huesca, Spain; 2 Applied Physics Department, Engineering Research Institute of Aragon (I3A), Faculty of Science, University of Zaragoza, Pedro Cerbuna 12, 50009 Zaragoza, Spain; juanval@unizar.es

**Keywords:** integrated Bragg grating, monolithic laser, active waveguide, critical parameters, integrated optics, erbium and ytterbium-codoped waveguides

## Abstract

Active waveguide Bragg gratings (AWBGs) are promising photonic structures that combine the very efficient reflective properties of a Bragg grating with the power amplification character of rare earths. This combination may lead to a potential monolithic laser under the proper conditions. However, the photonic response of these structures highly depends on the grating design and operating parameters, so modeling its response for their laser performance is a must. In this work, a numerical method is employed to calculate the optical power propagation along an Er/Yb-codoped integrated AWBG. A complete database of the AWBG response as a function of the most relevant operational parameters is obtained. As a result, the critical values of each design and operating parameter to achieve its laser performance have been determined, which represents very useful information for further experimental design, optimization, and fabrication of these photonic structures.

## 1. Introduction

Active waveguide Bragg gratings (AWBGs) are photonic structures in which a periodic perturbation of the refractive index is produced within a rare-earth-doped waveguide. On the one hand, a Bragg grating (BG) produces an efficient reflection of a light wave at those wavelengths that satisfy the Bragg condition [1]. This property lays the basis for several current and potential photonic applications in integrated optics, physical and chemical sensing, signal filtering, and laser cavities [2,3,4,5,6,7]. On the other hand, the rare earths’ active character may lead to light amplification [8,9]. Thus, both reflective and active properties are combined in an AWBG, leading to promising photonic applications and devices such as monolithic lasers and amplifying reflectors [10,11,12].

Ultrafast laser pulses technique for BG fabrication has been extended and intensified over the last decades and so has the experimental production, characterization, and optimization of these photonic structures [2,13,14,15]. Several kinds of uniform and non-uniform designs [3,6,16] and materials [17,18,19] have been considered, revealing a wide range of properties and applications. Furthermore, experimental works on AWBGs have been also developed over the last decade. AWBGs amplification [20] and laser performance [21,22], even in multimode/multicore lasers [23,24], have been achieved. However, the key point for the performance and operational range optimization of any potential AWBG-based photonic device is that both the passive BG and active medium responses are highly dependent on the design specifications, operating conditions, and even the manufacturing process itself. In this context, even some variables, such as the resulting material attenuation losses after the BG writing process, are far from a total precise control. This means that a minor variation in any of them could drastically modify the resultant AWBG response and, consequently, even cause or not the laser performance [25].

Some numerical models have been used in previous works to delve into these dependencies. Both the passive BG [26,27] and active waveguide [28,29] responses have been separately studied numerically. In addition, a few theoretical works have also been carried out on AWBGs [12,30]. However, an in-depth study of the fundamental dependencies and optimal ranges of the key AWBG parameters and operating conditions must be addressed to find the optimal designs and operational ranges for these promising photonic properties.

The aim of this work is to find the critical design and operating parameters of an AWBG that are necessary to achieve and optimize its laser performance. In order to do so, a complete numerical response database of Er/Yb-codoped integrated AWBGs is to be obtained as a function of the most relevant operational parameters. This will serve to study the optical power propagation along the AWBG as a function of each fundamental parameter in isolation but also to understand their collective contribution to the resultant AWBG performance. The AWBG response database will be useful for selecting and optimizing the design and operating parameters of these photonic structures for promising photonic applications such as monolithic lasing and amplifying reflectors.

The work is organized as follows. Section 2 describes the numerical method employed to simulate the power propagation throughout an AWBG. Section 3 presents the simulation results and discussions on the dependencies analyzed in isolation, as well as the critical values and operational ranges detected for the AWBG laser performance. Finally, Section 4 summarizes the principal findings and conclusions of the work.

## 2. Materials and Methods

### 2.1. Optical Power Propagation Along an AWBG

**Figure 1:**
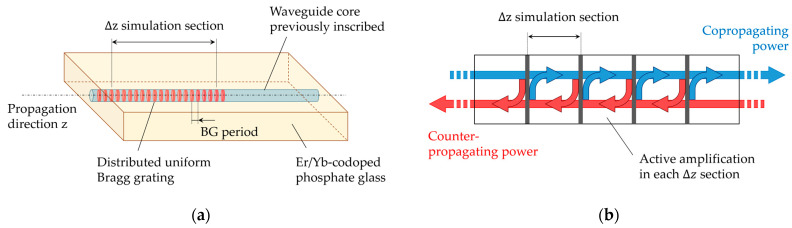
(**a**) Representation of a uniform Bragg grating inscribed in the Er/Yb-codoped waveguide, in which the difference between the BG period and the Δ*z* section considered for the numerical calculation is schematized (not to scale). (**b**) Representation of the multiple reflection behavior within each simulation section.

A numerical method previously developed by us [25] has been employed to simulate the optical power propagation evolution through an active Er^3+^/Yb^3+^-codoped integrated AWBG. In this structure, the BG would be written along the waveguide core previously inscribed in the bulk phosphate glass (Figure 1a). Since the typical BG period in this case is about 500 nm [25], simulating the periodic perturbations individually is not affordable. For this reason, the waveguide is divided into N small enough uniform sections of length Δz, each of which would contain about 100 grating periods (not to scale in Figure 1a).

Once the grating is divided, a Runge–Kutta method is employed to calculate the propagated power and its derivative in each section along the waveguide propagation direction z by means of the following equation:

(1) $$\frac{dP_{R/L}}{dz}=\left[A\left(\sigma ,\eta ,z\right)-\alpha +\frac{\ln\left(t\right)}{\Delta z}\right]P_{R/L}-\frac{\ln\left(t\right)}{\Delta z}P_{L/R}$$

where the subscripts *R* and *L* (right and left) refer to the co- and counterpropagating powers, respectively. The first term on the right of this equation considers the changes in the power, which propagates in the calculation direction. Within the brackets, three contributions are found. The first one, $A\left(\eta ,\sigma ,z\right)$, determines the Er/Yb active behavior along the propagation direction as a function of their transition cross-section, σ, and the overlapping factors between the mode intensity and population density distribution levels, η [28]. The second one, α, represents the medium’s power propagation loss coefficient. The third term considers the Bragg grating effect: A transmission coefficient t is assigned to each Δz grating section. Since the transmission coefficient is lower than one, the logarithm contributes negatively to the power derivative in the calculation direction, so that the reflected power turns to a negative change as logical.

On the other hand, the second term on this side of the equation considers a cross term: The fraction of power reflected by each grating section turns to increase the power that propagates opposite to the calculation direction, as observed in Figure 1b. This contributes positively to the power derivative, which is taken care of by the negative sign that modifies the logarithm one. This way, the fraction of co-propagated power reflected by each grating section turns to increase the counter-propagating power derivative, and vice versa. Therefore, both powers are mutually dependent on each other and a multiple-reflection behavior occurs. In addition, the active properties of the material might amplify the propagated power in each grating section, so that if this amplification overcomes the attenuation, each section starts to act as a laser cavity. Moreover, as each section transmits part of the generated power, they contribute to the surrounding ones, and, eventually, the grating as a whole works confining the light and leading to a laser behavior.

Taking into account the specific boundary conditions at both grating ends, an iterative calculation is carried out until a convergent solution is obtained. By means of this method, the pump and fluorescence powers (and the signal power, if such is the case) can be determined in each section considered and, as a consequence, the propagation evolution profile along the AWBG and the power emitted. In our simulations, a symmetric bidirectional pump power will be always injected at both grating ends (λ= 976 nm). The Er/Yb ions will turn some of it into fluorescence, with a peak in the Er^3+^ ion main amplification wavelength (λ= 1534 nm), inducing the laser cavity mechanism mentioned above.

### 2.2. Bragg Grating Specifications

The Bragg grating spectral response must be incorporated into the method. In previous works, the BGs experimentally written have shown a bandwidth below 1 nm [25]. Thus, the BG spectral response is simulated here by a delta function located at 1534 nm, corresponding to the Er^3+^ ion amplification peak. For that reason, simulations have been carried out within the wavelength range of 1525–1540 nm with a 1 nm resolution. This range has been previously selected and tested in order to obtain reliable results in affordable computational times. Thus, the transmission coefficient in Equation (1) will correspond to that of the grating section considered in each simulation at 1534 nm. At any other wavelength, the transmission coefficients are considered zero to simulate the delta function, so no reflective effects take place out of it.

The number of sections per unit length is aimed to be similar in all the simulations (20–30 sections/mm, as later detailed in Section 2.3). So, the total sections N in which it is divided is adjusted to the grating length in each case, and as a consequence, the transmission coefficient of each grating section has to be adjusted to obtain the same passive reflectivity value: $R_{o}=1-t^{N}$. In this work, the simulated AWBGs have uniform perturbations; so that the transmission coefficient is the same for every grating section to simulate the proper passive reflectivity at 1534 nm. The only information about the BG needed for the simulations is the transmission coefficient of this section. In the experimental AWBG fabrication process, the choice of the writing parameters such as the grating period, the periodic refractive index variation, and the medium refractive index is essential to ensure that the Bragg condition is satisfied at the Er^3+^ amplification wavelength [26]. However, this is only a manufacturing issue: Once the optimal designs have been identified and proposed for any experimental approach, the manufacturing details should be considered.

### 2.3. Iterative Calculation and Convergence Criterion

Changes in the power propagation after each iteration are small, especially at the beginning of the calculation, so simulations are performed in sets of 100 iterations. The convergence criterion for ending the iterative process is defined as follows. If the variation in both powers generated at the ABWG ends is less than 0.2%, and only if this occurs twice in a row (i.e., three consecutive sets), the solution is assumed convergent and the calculation stops. This double-check criterion has been chosen after several tests which showed that it usually leads to a convergence even below 0.01%. Some processes that would take much longer to reach this convergence value are avoided this way. Typically, a convergent solution is achieved after 2000–6000 iterations, depending on the AWBG.

On the other hand, in order to ensure the accuracy of the numerical calculations, a previous study on the required number of simulation sections, N, into which the grating is divided has been carried out. The numerical power generated at the grating ends has been compared in three representative $R_{o}$ values with four grating lengths. The mean deviations relative to 35 sections/mm are presented in Table 1.

As the number of sections/mm considered is increased, the deviation in the results asymptotically converges in all cases. However, the greater the number of sections, the longer the calculation time, maintaining typically a linear proportion. So, the number of sections must be chosen as a compromise between the reliability of the results and the feasibility in terms of computational time:

- The shorter the grating, the more sensitive the deviation is to the number of sections employed, as seen in Table 1. Using 35 instead of 30 sections/mm would increase the calculation time by about 17% (35/30), while the improvement in the response convergence would be only about 0.5%. Therefore, it seems acceptable to use 30 sections/mm for further simulations of AWBGs up to 5 mm in length, where simulation times are still affordable (about 16 h for each 5 mm simulation in our system).
- The same criterion in longer AWBGs implies a significant increase in the *N* number and, consequently, in calculation time. Considering the 10 mm case, using 30 instead of 20 sections/mm would increase the simulation time by a factor of 50% (30/20), while the results would only differ by 0.7%. The simulation time in such a case would be reduced from about 32 to 16 h. For this reason, for AWBGs longer than 5 mm, the number of sections has been chosen to be progressively reduced from 30 to 20 sections/mm as the length increases from 5 to 10 mm.

As explained in the following section, a large number of simulations have been carried out in this work. Although a high-capacity cluster has been used, which allows us to perform simultaneously up to 20 simulations, these criteria are fundamental for this work to be affordable. The validity of the numerical method and its considerations has been verified by a good theoretical-to-experimental agreement in our previous work [25].

## 3. Results and Discussion

The main objective of this work is to determine the critical values of the four fundamental grating and operating parameters of uniform AWBGs for their laser performance: pump power input, propagation losses, grating length, and grating passive reflectivity. In order to do so, several simulation sets have been carried out, in which a single parameter is modified. This will allow us to isolate the AWBG response dependencies on each fundamental parameter, as well as the critical conditions above which the laser performance of the structure is achieved.

### 3.1. Laser Regime as a Function of the Bragg Grating Reflectivity

**Figure 2:**
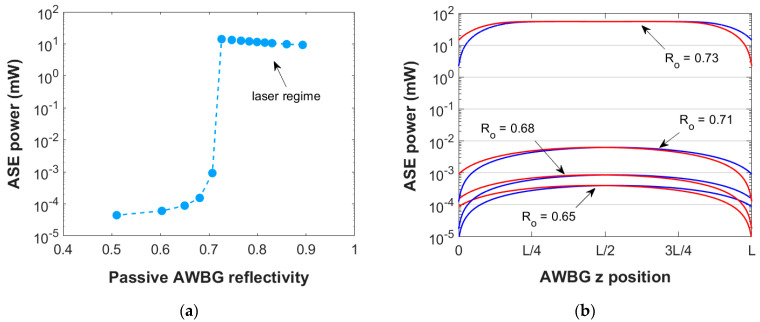
(**a**) Numerical results of the ASE power generated at 1534 nm by a 5 mm AWBG as a function of its passive reflectivity, under a bidirectional pump power input of 300 mW and a propagation loss coefficient of 1.43 dB/cm; (**b**) ASE power profiles along the AWBG propagation direction with passive reflectivities near the critical value to enter the laser regime. Co- and counterpropagating powers are shown in blue and red, respectively.

Figure 2a presents the numerical results of the ASE power generated at 1534 nm by a 5 mm Er/Yb-codoped AWBG as a function of its passive reflectivity. In order to isolate this dependence, the propagation loss coefficient and pump power employed in these simulations are held constant: 300 mW and 1.43 dB/cm, respectively. This propagation loss coefficient is within the normal range for this type of material [14]. In the case of low grating passive reflectivity, the generated ASE power is almost negligible (0.1–1 µW). However, as the BG reflectivity is increased, the ASE power starts increasing until a sharp change occurs: The ASE power increases four orders of magnitude and the lasing regime is achieved. This effect can be better understood by analyzing the amount of ASE power propagated within the AWBG around the transition reflectivity value (Figure 2b): The grating increasingly confines the optical power and it gets drastically amplified. The grating enters a self-amplified multiple reflections regime, and the ASE power obtained at the grating end is about 14 mW.

Above this critical reflectivity, the lasing behavior still occurs (Figure 1a), at least in the reflectivity range simulated. Nevertheless, the power generated decreases slightly since the more reflective each grating section is, the more difficult it becomes for the optical power to spread throughout the entire grating, so the total active effect is reduced. Eventually, as the grating passive reflectivity approaches one, that is to say, no power enters the grating, it would be expected that there would appear an upper critical point beyond which the laser regime would not be achieved: The optical power could not propagate along the grating. However, gratings of such reflectivity (above R = 0.9) are not of interest to this study since they represent extremely high values to be reached in the experimental fabrication procedure.

### 3.2. Dependences on the Other Fundamental AWBG Parameters

A complete set of simulations similar to Figure 2 has been carried out varying the other three parameters (propagation losses, pump power, and grating length) in order to find the critical point observed before at which the AWBG enters the laser regime in each case.

#### 3.2.1. Pump Power

**Figure 3:**
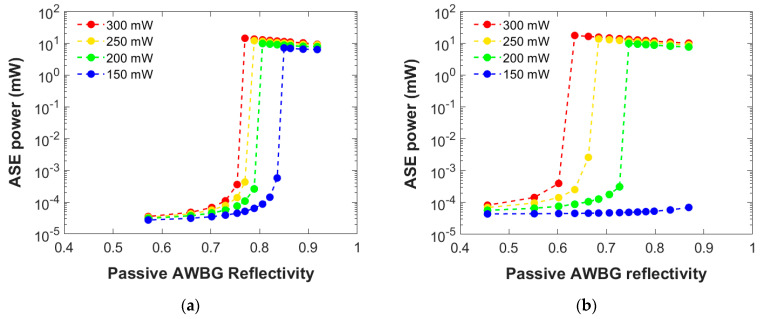
Numerical results of the ASE power generated at 1534 nm by a (**a**) 4 mm and (**b**) 8 mm AWBG as a function of its passive reflectivity and the bidirectional pump power injected. A propagation loss coefficient of 1.30 dB/cm is employed in this simulation set.

Figure 3 presents the numerical results of the ASE power generated at 1534 nm by a 4 and 8 mm AWBG as a function of its passive reflectivity considering different pump powers symmetrically injected to both grating ends (a loss coefficient 1.30 dB/cm is held constant). As observed, the lower the pump power employed, the more reflective the Bragg grating is needed in order to achieve the laser regime. If the pump power is not enough, as such is the case with 150 mW in Figure 3b, this behavior is not observed within the entire simulated reflectivity range. So, depending on the pump power injected, a minimum reflectivity value is needed to reach the laser performance.

The dependence observed is logical: The lower the pump power, the lower the amplification by the active materials. The only way to compensate for this shortage is by increasing the Bragg grating reflectivity in order to favor the optical power confinement and self-amplification near the pump power entrances and later extend to the rest of the AWBG.

Nevertheless, as the pump power employed is increased, the critical value of reflectivity required in the 8 mm AWBG starts to move towards lower reflectivities faster than the 4 mm AWBG, and, eventually, a lower reflectivity is needed: 0.635 for the 8 mm AWBG with 300 mw versus 0.770 for the 4 mm one. The reason for this trend inversion is simple: When the pump power is enough to reach the grating far from each entrance, the laser behavior may occur throughout the entire AWBG. In that case, the longer the grating, the more active the material capable of generating ASE power, so less BG reflectivity is needed to achieve the critical point for its performance. However, this also means that the longer the grating, the more sensitive it is to the pump power value, so the range of this operating parameter becomes more restricted as clearly observed in comparison in Figure 3.

#### 3.2.2. Propagation Losses

**Figure 4:**
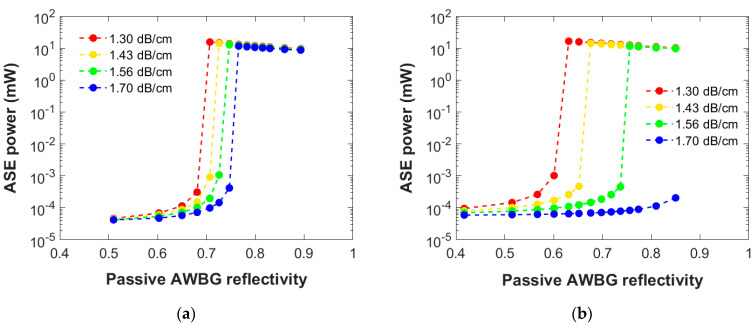
Numerical results of the ASE power generated at 1534 nm by a (**a**) 5 mm and (**b**) 10 mm AWBG as a function of its passive reflectivity and the propagation loss coefficient. A 300 mW bidirectional pump power is employed in this simulation set.

Figure 4 presents the numerical results of the ASE power generated at 1534 nm by a 5 and 10 mm AWBG as a function of its passive reflectivity considering different propagation losses within an acceptable range for these materials [14] (a 300 mW pump power is held constant). A comparable but opposite dependence is observed regarding the attenuation: As it impairs the optical power propagation, the higher the losses, the more reflective the AWBG must be in order to reach the laser behavior. Therefore, considering a grating whose reflectivity allows us to achieve the laser regime within this range of losses is essential for any further experimental design and manufacture.

A similar dependency to that observed in Figure 3 occurs: Depending on the propagation losses, a minimum reflective value is needed for the laser regime. Again, the laser performance might not be observed above a certain loss value, as it occurs in Figure 4b with 1.70 dB/cm. Moreover, this result can be extended to different pump powers, making the laser performance more difficult to achieve as the pump power used becomes lower, as seen before.

For the same reason as mentioned in Figure 3, the minimum value required in a long grating with low propagation losses is lower than in a short one: 0.631 for the 10 mm AWBG with 1.30 dB/cm versus 0.707 for the 5 mm one. In that case, the longer the grating, the more active the material is working to generate the ASE power. However, this also means that the longer the grating, the more sensitive it is to the propagation losses, so the range of this operating parameter is more restricted. This result is highly important since the grating propagation losses after experimental fabrication are not a parameter that is easy to control. Thus, the optimal design to be considered for fabrication should be able to operate over a sufficiently wide propagation loss range to ensure its functionality.

#### 3.2.3. Grating Length

The dependence on the AWBG length is not as obvious as those previously analyzed since opposite contributions are mixed. On the one hand, the propagation losses in an optical medium increase exponentially with the material length, $e^{-\alpha L}$, so the longer the AWBG, the more attenuated the optical power will be. On the other hand, if the pump power employed is enough, the rare earths’ active character can compensate for these losses and eventually lead to high amplification. As already discussed before, the longer the grating, the more active the material and the more total amplification could be induced. So, it is logical to check how this aspect affects the minimum AWBG passive reflectivity required for the laser behavior regarding the grating length considered.

**Figure 5:**
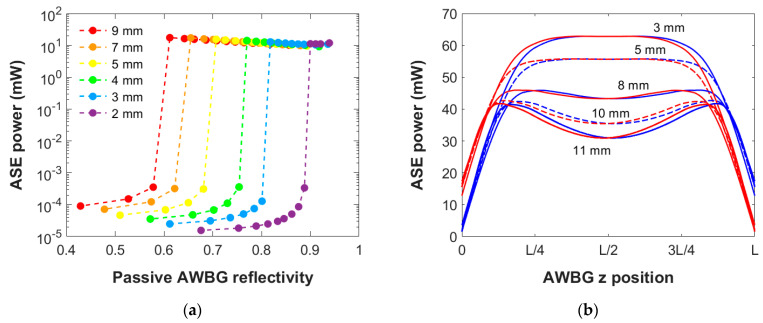
(**a**) Numerical results of the ASE power generated at 1534 nm by AWBG as a function of its passive reflectivity and the grating length. A 300 mW bidirectional pump power and 1.30 dB/cm propagation loss coefficient are employed in this simulation set. (**b**) ASE power profile along the AWBG propagation direction in several grating lengths with the minimum reflectivity for the transition to laser regime in each case. The *z* scale is rescaled to each grating length *L* for a better comparison. Co- and counterpropagating powers are shown in blue and red, respectively.

Figure 5a presents the numerical results of the ASE power generated by AWBGs in the range of 2–9 mm length under the same pump power and propagation loss conditions: 300 mW and 1.30 dB/cm. As observed in this range, the longer the grating, the lower the critical passive reflectivity needed to achieve the laser behavior. This result matches the above-mentioned argument: The more active the material working, the more amplification and so less power confinement is required. Using a 9 mm AWBG would lead in this specifications to the laser regime with a grating of only about $R_{o}=$ 0.612, which is a value quite affordable experimentally [25]. However, a 2 mm AWBG would require a passive reflectivity $R_{o}=$ 0.90, which is an excessively high value for its experimental fabrication. It should be noted that the maximum reflectivity of a passive Bragg grating (without considering propagation losses) is determined by $R_{o,max}=\tanh^{2}\left(\kappa L\right)$, being κ the BG coupling coefficient which describes the grating reflective efficiency [26]. Therefore, the longer the grating, the lower the coupling coefficient needed, making it easier to fabricate. Since it is not the objective of this work, we always refer, in this work, to the final BG passive reflectivity instead of the coupling coefficient (obviously the specifications for it should be taken into consideration in its manufacture [26]).

Nevertheless, the trend observed in Figure 5a regarding the critical reflectivities needed as a function of the length becomes less significant as the AWBG length increases. In fact, the numerical results obtained with a 10 mm grating have indicated us that a critical $R_{o}=$ 0.631 is needed under the same conditions of this simulation set, which is higher than that with 9 mm: $R_{o}=$ 0.612 (the 10 mm curve is not presented in Figure 4a for a sake of clarity). The reason for this trend inversion lies in the fact that the grating ends, where the pump power is injected, are farther apart. As seen in the ASE power profile along the AWBG, Figure 5b, as the grating simulated is longer, the ASE power generated near the entrance does not extend far enough across the entire grating. In that case, the ASE power profile starts forming two peaks along the grating, in which the light is becoming more confined near the grating ends (where the pump power is injected), but reduced in the grating center. Thus, the AWBG does not operate as efficiently as possible. As a result, above a length of about 9 mm, a more reflective grating is required in order to increase the multiple reflections and the power confinement within the AWBG for its laser performance to compensate for this shortage. This inversion trend is numerically observed in Table 2 where, for the same reason, an inversion is also seen in the ASE power generated at the AWBG end as a function of its length.

These results correspond to a single combination of pump power and propagation losses, in which an optimal 9 mm design would minimize the required BG reflectivity leading to the maximum ASE power generation. Nevertheless, the result will logically depend on the parameters combination. Therefore, a detailed analysis of all these results and combinations is required in order to adequately select the parameters for any further optimal experimental design.

### 3.3. Critical Values for Laser Performance

Once all the previous simulations have been carried out, a complete AWBG response database has been obtained for all combinations of the four fundamental parameters analyzed (up to more than 2200 combinations). The four-parameter ranges simulated are indicated in Table 3. Regarding the reflectivity, the starting and ending values are adjusted depending on the grating length (the longer the grating, the lower the starting reflectivity, as seen in Figure 5a) in order to minimize the total calculation time and ensure that the transition point is observed in each case (if such is the case). As a consequence, the critical reflectivity needed for every design and working condition is found when the laser regime is achieved within the simulated ranges. This critical value has been chosen as the minimum passive reflective in which the transition to laser regime is obtained in our simulation set, as shown in Table 2.

**Figure 6:**
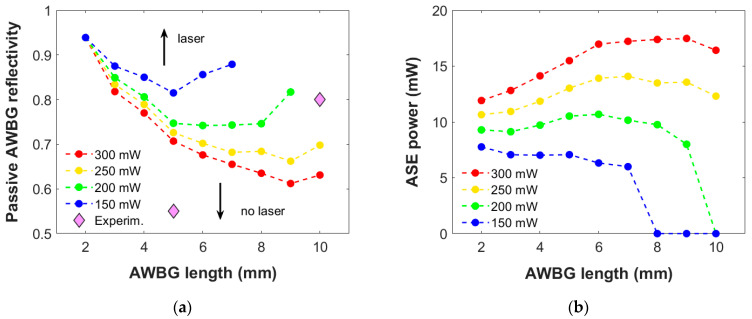
(**a**) Critical AWBG passive reflectivity for the transition to the laser regime as a function of the grating length and pump power injected. Two experimental values are indicated with a diamond-shaped dot. (**b**) ASE power is generated in those AWBGs just above the transition to the laser regime. A propagation loss coefficient of 1.30 dB/cm is employed.

Figure 6a presents the critical passive AWBG reflectivity needed to enter the laser regime as a function of its length and the pump power injected with a 1.30 dB/cm loss coefficient. Below these reflectivity values, AWBG cannot reach the laser behavior in this case and pump powers. As observed in these results, a long design will allow the AWBG to work in the laser regime with passive reflectivity values lower than 0.8, which is an acceptable value for its experimental fabrication [25]. However, the longer the grating, the more sensitive to the pump power employed: No laser behavior is detected under 200 mW with 10 mm and under 150 mW with gratings longer than 7 mm. On the contrary, the shorter the AWBG, the more similar the critical passive reflectivity, so that the same design would allow to achieve the laser regime within the pump power range simulated. Nevertheless, writing such a small and efficient BG is a highly difficult task: The BG passive reflectivity depends on its length as mentioned in Section 3.2.3.

In Figure 6a, an upper critical reflectivity value could be expected above which the grating would be too reflective to allow the adequate propagation of the optical power propagation along the entire waveguide. However, this is not the aim of this study since the passive reflectivity values would be too high for any further experimental manufacture, as mentioned before.

On the other hand, Figure 6b presents the ASE power generated by the AWBG at the transition conditions. As expected, the ASE power in each grating length grows proportionally with the pump power injected while it is enough to enter the laser regime. Moreover, maximum power is achieved under the same pump power and propagation loss conditions, as discussed in Table 2. However, this point occurs in different lengths for each pump power input: The optimal 9 mm design mentioned in Section 3.2.3 would not enter the laser regime with a 150 mW design, so it would be restricted to a narrower working conditions range.

These numerical results have been compared with two experimental AWBGs manufactured by us [25]. On the one hand, a 10 mm AWBG was written with $R_{o}=0.80$ and characterized, showing a small but significant ASE power when using over 220 mW pump power. On the other hand, a 5 mm AWBG was written with $R_{o}=0.55$, and no significant ASE power was detected for any pump power employed in this same range. Both experimental results are presented with a diamond-shaped dot in Figure 6a. A notable agreement is found between these experimental results and the simulated critical values. The experimental 5 mm AWBG was designed under the critical values, and therefore, no laser behavior would be expected. The experimental 10 mm result is found above the simulated critical value, so a laser regime would be expected in that case depending on the pump power: experimentally observed only over 220 mW, which matches the fact that it was not observed with 200 mW. It also confirms that the propagation loss range considered for this type of material is consistent. These theoretical-to-experimental comparisons validate our numerical method but also represent clear evidence of this work’s usefulness in considering an adequate design and conditions before manufacturing the AWBG.

**Figure 7:**
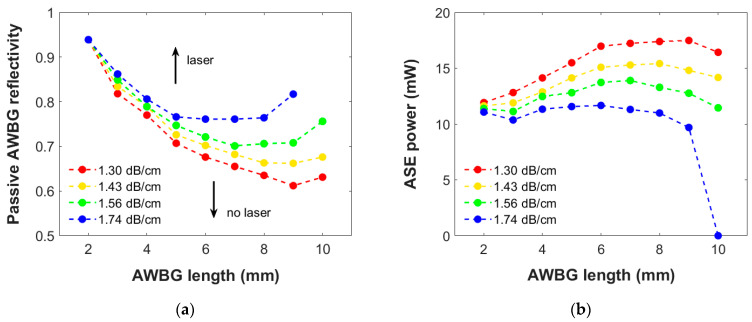
(**a**) Critical AWBG passive reflectivity for the transition to the laser regime as a function of the grating length and propagation loss coefficient; (**b**) ASE power generated in those AWBGs just above the transition to the laser regime. A bidirectional pump power of 300 mW is employed.

Moreover, Figure 7 presents the same results but concerning the loss coefficient under a single pump power of 300 mW. The results in Figure 7a show similar behaviors: The shorter the grating, the more efficient the BG is needed, as already mentioned in Section 3.2.2, and a trend inversion is again observed in both dependencies. This comparison highlights the importance of the propagation losses to the laser performance: A 9 mm AWBG would require a passive $R_{o}$ from 0.61 to 0.82 depending on the final losses’ range considered, which represents a substantial difference in its manufacturing design. Since the AWBG propagation losses are not easy to be controlled in the writing process, this is a key factor to take into account since the experimental structure can present an attenuation within this typical range. On the other hand, the shorter the grating, the less sensitive the design will be to the propagation losses range, but it would require too high reflectivity values (above 0.8).

On the other hand, as shown in Figure 7b, the ASE power generated by the AWBG at the transition conditions under the same pump power is clearly reduced as the considered losses are increased. As expected, this behavior becomes more significant as the grating is longer. However, it is worth noting that the observed deviation can vary up to 7–8 mW, which is a very significant variation. This result highlights the fundamental need to have sufficient control in the manufacturing process over the losses of the final structure in order to optimize its response for any potential application.

## 4. Conclusions

A numerical method has been employed to simulate the power propagation along an Er/Yb-codoped integrated AWBG as a function of the four most relevant parameters, which determines its photonic response: grating length, pump power input, propagation losses, and Bragg grating reflectivity. This has allowed us to examine the AWBG response dependencies on each one of them in isolation to find the critical values for the grating to enter the laser regime. As a result, a complete AWBG response database has been generated departing from the simulation of each possible combination of these fundamental parameters within their typical ranges in these materials. The operational ranges of each possible BG have been identified over which the AWBG laser regime would be achieved. Furthermore, the sensitive variation of the AWBG response to any minor parameter variation has revealed the ranges of these parameters, which lead to the AWBG laser performance.

In particular, in terms of the potential propagation loss contribution, very significant differences have been found within the loss range usual for these photonic structures after their manufacturing process. Given that the control of these losses in such fabrication techniques is very complex, even sometimes somewhat random, the applicability of this work is enhanced to ensure the design that could optimize and extend the operating range in any potential lasing application.

Finally, the numerical results have been compared with those of two previous experimental AWBGs with completely different characteristics and responses. This comparison has shown a good agreement below and above the critical values obtained numerically, which verifies the consistency of the numerical method. Therefore, the usefulness of the developed AWBG database for a proper selection of the BG according to the operating conditions is again highlighted. This information would be very representative of the further experimental design, optimization, and fabrication of these photonic structures in view of potential applications such as robust monolithic lasers, optical sensors, and amplifiers or active mirrors in more complex inscribed cavity lasers.

## Figures and Tables

**Table 1:** Mean deviation of the numerical calculation as a function of the sections/mm relative to the results obtained with 35 sections/mm. The deviation is compared across four AWBG lengths. The number N of total sections (and its value per unit length) chosen for further simulations is given.

Sections/mm	3 mm	5 mm	7 mm	10 mm
30	0.5%	0.4%	0.3%	0.3%
25	1.1%	0.8%	0.7%	0.6%
20	2.1%	1.5%	1.3%	1.0%
15	3.6%	2.8%	2.4%	2.1%
10	6.0%	5.0%	4.2%	3.8%
**Chosen N (sections/mm)**	90 (30)	150 (30)	170 (24.3)	200 (20)

**Table 2:** Minimum AWBG passive reflectivity needed to enter the laser regime in Figure 5 results as a function of the AWBG length, and ASE power generated at the end of the grating. The 9 mm AWBG’s ASE power profile is not presented in Figure 5b to better compare the peak evolution.

AWBG Length (mm)	Minimum *R_o_*	ASE Power (mW)
3	0.818	12.8
5	0.707	15.5
8	0.635	17.4
9	0.612	17.5
10	0.631	16.4
11	0.635	15.6

**Table 3:** Ranges of the fundamental design and working parameters studied in the simulations.

Parameter	From	To	Steps
Pump power (mW)	150	300	50
Length (mm)	2	10	1
Reflectivity	0.40/0.67	0.85/0.95	Variable
Losses coefficient (dB/cm)	1.30	1.74	Variable

## Data Availability

Data are available on request.
